# The Impact of Pembrolizumab as a Salvage Therapy Based on HER2 Expression in Advanced Gastric Cancer

**DOI:** 10.3390/cancers16172969

**Published:** 2024-08-26

**Authors:** Sung Hee Lim, Min Jung Kim, Jeeyun Lee, Ho Yeong Lim, Won Ki Kang, Seung Tae Kim

**Affiliations:** Division of Hematology-Oncology, Department of Internal Medicine, Samsung Medical Center, Seoul 06351, Republic of Korea; sunghee1022.lim@samsung.com (S.H.L.); mjkds2102@sch.ac.kr (M.J.K.); jyun.lee@samsung.com (J.L.); hoy.lim@samsung.com (H.Y.L.); wonki.kang@samsung.com (W.K.K.)

**Keywords:** gastric cancer, pembrolizumab, HER2 expression

## Abstract

**Simple Summary:**

This study evaluated the effectiveness of salvage pembrolizumab in advanced gastric cancer (AGC) patients based on HER2 expression. Conducted at Samsung Medical Center from November 2017 to March 2023, it involved 113 patients treated with pembrolizumab. Twelve patients (10.6%) were HER2-positive, and 101 were HER2-negative. Results showed no complete responses among 92 evaluable patients. However, 50% of HER2-positive patients had a partial response compared to 4.9% of HER2-negative patients (*p* < 0.001). Disease control rates were 70% for HER2-positive and 37.8% for HER2-negative patients (*p* = 0.086). Median progression-free survival was 5.53 months for HER2-positive patients versus 1.81 months for HER2-negative patients (*p* = 0.037). This indicates that salvage pembrolizumab monotherapy is more effective in HER2-positive AGC patients.

**Abstract:**

Immune checkpoint inhibitors (ICIs) are used as salvage treatments for advanced gastric cancer (AGC) regardless of HER2 status. This study assessed the efficacy of ICIs based on HER2 expression in AGC patients who received pembrolizumab as salvage monotherapy at Samsung Medical Center from November 2017 to March 2023. HER2 status was determined via immunohistochemistry, and tumor response and survival outcomes were compared accordingly. Among the 113 patients analyzed, with a median age of 61 years and 64.6% being male, 12 patients (10.6%) were HER2-positive, and 101 patients (89.4%) were HER2-negative. Of 92 evaluable patients, none had a complete response. However, 50% of HER2-positive patients had a partial response, compared to 4.9% of HER2-negative patients (*p* < 0.001). The disease control rate was 70% in HER2-positive and 37.8% in HER2-negative patients (*p* = 0.086). Median progression-free survival was 5.53 months for HER2-positive patients versus 1.81 months for HER2-negative patients (*p* = 0.037). Pembrolizumab as a salvage chemotherapy for the treatment of AGC demonstrated superior effectiveness in HER2-positive patients compared with HER2-negative patients.

## 1. Introduction

Gastric cancer is the fourth leading cause of cancer deaths, with approximately 0.8 million deaths annually [1]. Although various chemotherapeutic agents have been applied to treat metastatic advanced gastric cancer (AGC), disease outcomes remain poor. Most patients with AGC do not benefit from molecularly targeted agents, unlike those with non–small cell lung cancer or colorectal cancer. Expected progression-free survival (PFS) and overall survival of newly diagnosed AGC patients are devastating, at 5.2 to 7.7 months and 12.4 to 14.1 months, respectively, even with the use of immune checkpoint inhibitors (ICIs) in the first-line setting [2,3,4,5].

Approximately 20% of advanced gastric or gastroesophageal junction adenocarcinomas have human epidermal growth factor receptor 2 (ERBB2, or HER2) amplification or overexpression [6]. In some studies, a positive result on an HER2 test has been associated with poor prognosis [7,8,9,10], while others have suggested that an HER2-positive status itself is not an independent prognostic factor [11,12]. Unlike breast cancer, the impact of HER2 positivity on the prognosis of AGC has yet to be clearly established. Despite this uncertainty, adding trastuzumab to HER2-positive AGC treatment has been shown to provide unambiguous overall survival benefits [13].

More recently, combining pembrolizumab with trastuzumab and chemotherapy for HER2-positive gastric cancer resulted in a further improvement in objective response rates (ORRs) of 74.4% versus 51.9% in a trastuzumab-plus-chemotherapy study arm [14]. The combined use of ICIs and trastuzumab reportedly strengthens the immune response of HER2-specific T cells, promotes immune-cell trafficking, and triggers an expansion of peripheral memory T cells [14]. This synergy between HER2 blockades and programmed cell death protein-1 (PD-1) inhibition is well established [15,16,17], but little attention has been paid to the association between HER2 overexpression and PD-1 inhibition. Here, we investigate whether the efficacy of PD-1 inhibitor monotherapy as a salvage therapy differs depending on the status of HER2 expression.

Pembrolizumab is a potent, highly selective, IgG4-κ humanized monoclonal antibody that prevents PD-1 binding with PD-L1 and PD-L2. It was generated by grafting the variable region sequences of a mouse antihuman PD-1 antibody onto a human IgG4-κ isotype framework containing a stabilizing S228P Fc mutation [18]. Under normal circumstances, PD-1 binds with its ligands, PD-L1 and PD-L2, found on cancer cells and other cells in the tumor microenvironment, leading to the inhibition of T cell activity. This interaction helps tumors evade the immune system. Pembrolizumab blocks this binding, preventing PD-1 from interacting with its ligands, thereby reactivating T cells. This reactivation enhances the immune system’s ability to recognize and destroy cancer cells, leading to improved anti-tumor responses in patients [19].

## 2. Materials and Methods

### 2.1. Patients

We analyzed AGC patients receiving pembrolizumab monotherapy as a salvage therapy between November 2017 and March 2023 at the Samsung Medical Center. HER2 expression was evaluated by immunohistochemistry (IHC) tests. Patients with pathologically confirmed gastric adenocarcinoma were included, and patients without available HER2 immunohistochemistry results were excluded. Clinical characteristics, including age, sex, and Eastern Cooperative Oncology Group performance status, disease status, HER2 and programmed cell death-ligand 1 (PD-L1) status, Epstein–Barr virus in situ hybridization, and microsatellite instability (MSI) results from patients’ tissue samples, were collected and reviewed. The programmed cell death-ligand 1 (PD-L1) status of tumor samples was evaluated by pharmDx immunohistochemistry assays (PD-L1 IHC 22C3; Agilent Technologies, Santa Clara, CA, USA). The study protocol was approved by the Institutional Review Board of the Samsung Medical Center (Seoul, Republic of Korea). The study was conducted in compliance with the Declaration of Helsinki.

### 2.2. HER-2 (c-erbB-2) Immunohistochemistry Test

HER-2 tests were performed by IHC, using a VENTANA anti-HER2/neu rabbit monoclonal primary antibody (clone 4B5, VENTANA Medical System, Tuscon, AZ, USA), at the Department of Pathology, Samsung Medical Center, in Seoul. The IHC test results for HER-2 were interpreted according to the HERACLES diagnostic criteria [20], with a positive result defined as an intensity score of 3 or greater, which indicates this intensity in more than 10% of the cells. 

### 2.3. Statistical Analysis

We used descriptive statistics to summarize patient characteristics. For nonparametric comparisons of continuous variables, we employed Mann–Whitney U tests. Fisher’s exact tests were used to compare categorical variables, including sex, disease characteristics (such as HER2 status and PD-L1 status), overall response rates (ORRs), and disease control rates (DCRs). Tumor response was evaluated as complete response (CR), partial response (PR), stable disease (SD), or progressive disease (PD), according to the Response Evaluation Criteria in Solid Tumors version 1.1 (RECIST 1.1). DCR was defined as the percentage of patients with confirmed CR, PR, or SD. PFS was measured from the onset of administration of the ICI to the date of disease progression or death from any cause using RECIST 1.1. Overall survival was calculated from the start of the ICI to the date of death from any cause. We applied the Mann–Whitney U test to test the difference in nonparametric variables of the two groups. Kaplan–Meier curves with log-rank tests and Breslow’s method were used for survival analysis. We used SPSS version 29.0 for statistical analysis and R version 4.3.0 for statistical analysis and data visualization.

Response and survival outcomes were assessed with propensity matching. We used a matching algorithm to match HER2-negative and HER2-positive patients at a 2:1 ratio based on age without replacement or calipers. We checked the imbalance after matching. We conducted Fisher’s exact tests for ORR and DCR. We also estimated survival results by using Kaplan–Meier’s method and a Cox proportional hazards regression model.

## 3. Results

### 3.1. Patient Characteristics

A total of 113 patients were included in the study (Table 1). Among them, 12 (10.6%) were HER2-positive, and 101 (89.4%) were HER2-negative. The median age of the entire study population was 61 (range, 32–84), and 73 patients (64.6%) were male. Among the 45 patients with an available PD-L1 combined positive score (CPS), 33 patients (73.3%) had a PD-L1 CPS of 1 or higher. Two patients had high MSI diseases, representing 2.0% of the 101 patients with available MSI tests. The median number of patients with prior palliative chemotherapy was 3 (range 2–5), which indicates the heavily treated history of our study population. There was no difference in previous palliative chemotherapy lines between the HER2-positive and HER2-negative group. Patient characteristics are summarized in Table 1. There was no difference in baseline characteristics between the two groups, except for age. The median age of HER2-positive patients was higher than that of HER2-negative patients (*p* = 0.005). Of the total 113 patients, 81 (71.7%) had metastatic gastric cancer from diagnosis and 32 had recurrence after curative surgery.

### 3.2. Treatment Efficacy

Table 2 presents the objective responses to pembrolizumab as salvage chemotherapy. Among the 92 evaluable patients, 9 achieved a PR, representing an ORR of 9.8%. In the subgroup analysis based on HER2 overexpression status, 5 of 10 HER2-positive patients (50%) and 4 of 82 HER2-negative patients (4.9%) had a PR (*p* < 0.001). The DCR was 70% in HER2-positive patients and 37.8% in HER2-negative patients (*p* = 0.086). The unadjusted odds ratio of the ORR of HER2-positive to HER2-negative was 19.5 (95% confidence interval (CI), 3.954 to 96.168; *p* < 0.001), while the age-adjusted odds ratio was 13.008 (95% CI, 2.255 to 75.023; *p* = 0.004).

### 3.3. Progression-Free Survival and Overall Survival

**Figure 1:**
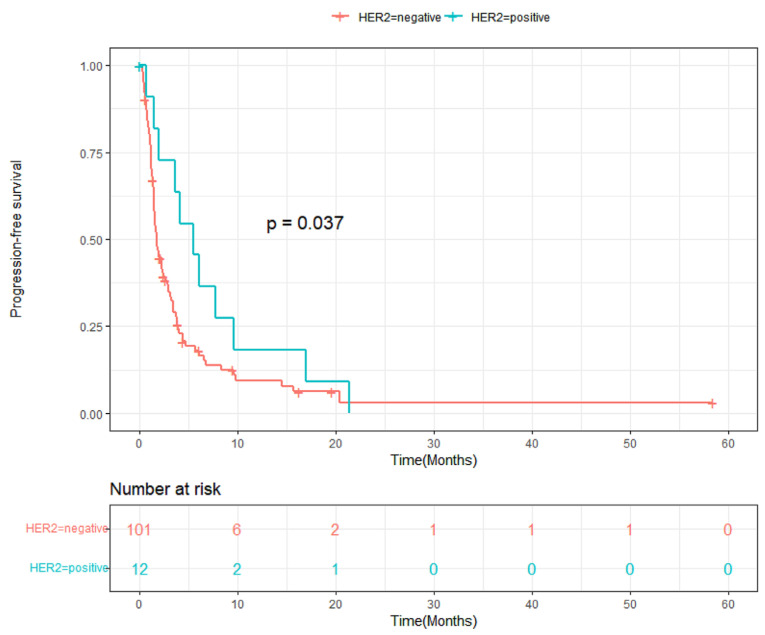
Kaplan–Meier curve for progression-free survival in HER2-positive and HER2-negative patients. *p*-value calculated with Breslow’s method.

**Figure 2:**
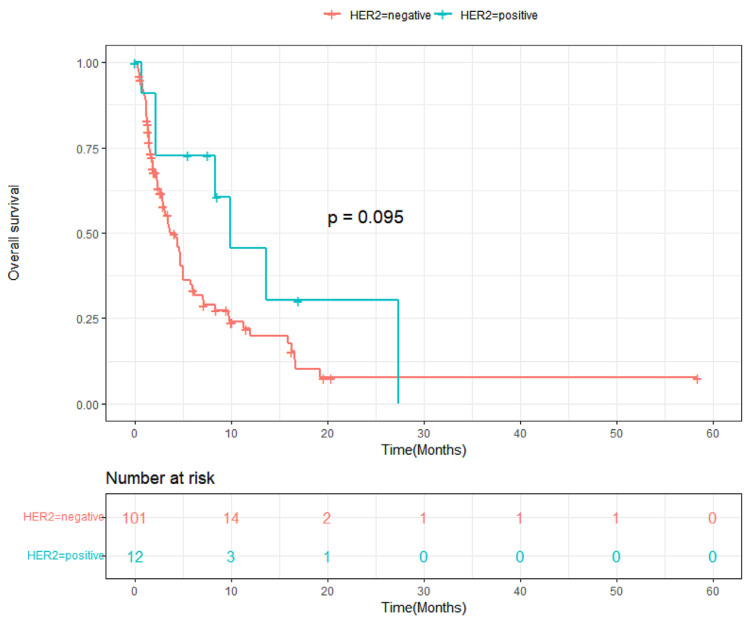
Kaplan–Meier curve for overall survival in HER2-positive and HER2-negative patients. *p*-value calculated with Breslow’s method.

**Figure 3:**
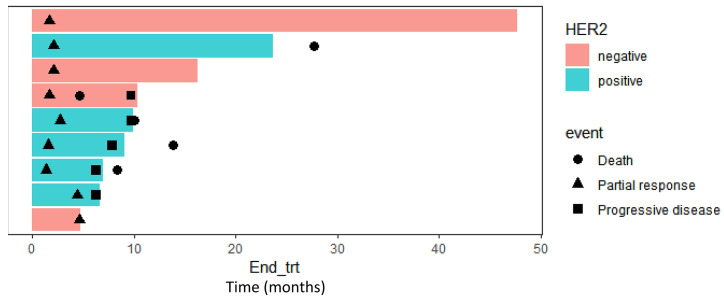
Swimmer’s plot of HER2-positive and HER2-negative patients who were confirmed as having a partial response.

The HER2-positive patients exhibited a median PFS of 5.53 months (95% CI, 2.90 to 8.15), which was significantly longer than that of the HER2-negative patients (median 1.84 months; 95% CI, 1.48 to 2.21; *p* = 0.037) (Figure 1). The median overall survival (OS) was 9.97 months (95% CI, 3.89 to 16.05) in HER2-positive patients and 3.65 months (95% CI, 2.21 to 5.09) in HER2-negative patients, with a hazard ratio of 0.609 (95% CI, 0.277 to 1.337; *p* = 0.095) (Figure 2). A swimmer plot showing the response duration of responders was shown in Figure 3, and it appears longer in HER2-positive patients.

## 4. Discussion

Pembrolizumab monotherapy achieved a superior tumor response in HER2-positive patients compared with HER2-negative patients. Significant differences in PFS were also evident between the two groups. These findings suggest that pembrolizumab monotherapy can be considered as salvage therapy for HER2-positive gastric cancer patients.

For HER2-positive gastric cancer patients, although combination therapy with trastuzumab and chemotherapy provides meaningful survival benefit [13], many clinical trials targeting HER2 have failed to demonstrate clinical benefits in subsequent-line treatment [21,22,23]. Trastuzumab deruxtecan, an antibody-drug conjugate, has shown positive results even in later-line treatment and has become the new standard treatment [24]. Looking at the effects of immune checkpoint inhibitors in later-line treatment for AGC, in a phase 2 study of patients with AGC whose disease had progressed after two or more lines of therapy, pembrolizumab was effective in some patients, with an ORR of 11.6% [25]. Nivolumab monotherapy also exhibited promising efficacy in recurrent or metastatic gastric cancer patients who were refractory to, or intolerant of, two or more previous chemotherapies [26]. The median OS was longer in the nivolumab group than in the placebo group (5.26 months versus 4.14 months) and the ORR was 11.2% [26].

Although a small proportion of AGC patients are responsive to ICIs, predictive biomarkers with confirmed evidence are rare. In gastric cancer, the ORR of pembrolizumab monotherapy was not correlated with PD-L1 CPS [25]. Recently, studies have been designed to define predictive models for ICI responses in solid cancers, using not only clinical features but genetic and molecular markers as well [27,28,29,30,31]. However, only a small portion of the analyzed samples are gastric cancer, and they have yet to be rigorously validated. Although our study and similar others are limited by a small sample size, particularly a limited number of HER2-positive patients, in this study, we suggest the possibility of using HER2 as a simple biomarker to predict responses after taking pembrolizumab in patients with AGC.

In the present study, the median age of HER2-positive patients was higher than that of the HER2-negative group. Limited evidence exists regarding the correlation between age and HER2 status, but some retrospective studies suggest older patients may have higher HER2 positivity rates [30,31,32,33]. In a retrospective observational study that analyzed the age variation among HER2 IHC-positive patients in 27,787 biopsy specimens of gastric cancer, the rates were 7.1%, 8.1%, 9.0%, 10.9%, 11.8%, 12.6%, and 12.1% for patients aged ≤30, 31–40, 41–50, 51–60, 61–70, 71–80, and >80 years, respectively (*p* < 0.001) [30]. In another retrospective study evaluating the frequency of HER-positive cases in patients with resectable gastric cancers, 25 of 213 patients were HER2-positive. While the mean age of HER2-negative patients was 66.2, that of HER2-positive patients was 71.2 (*p* = 0.0309) [31]. In another study, while 346 of 744 patients (46.5%) with HER2-negative GC were older than 59, a total of 63 of 94 patients (67.0%) with HER2-positive gastric cancer were older than 59 (*p* < 0.001) [32]. The HER2-positive rate in patients ≤59 years old was 7.2%, while the HER2-positive rate in patients older than 59 years was 15.4% (31 out of 429 and 63 out of 409, respectively). Interpreting the data without adjusting for age may therefore be more effective if advanced age in the HER2-positive group is a natural epidemiologic feature.

In the HER2-positive patient group, all patients received trastuzumab plus chemotherapy as their first-line treatment. Before starting pembrolizumab, 9 (75%) patients were treated with third-line chemotherapy and 3 (25%) patients were treated with second-line chemotherapy. In this study, none of them received trastuzumab deruxtecan before pembrolizumab. Therefore, anti-HER2 therapy was not used immediately before pembrolizumab. Prior use of trastuzumab might influence the efficacy of pembrolizumab, but since they were not used simultaneously or immediately sequentially, it is difficult to fully explain the mechanism. 

There is no definitive evidence to explain the high response rate in HER2-positive cases. In our opinion, there may be a synergistic effect in HER2-positive patients previously treated with trastuzumab as the drug has a relatively long half-life of 28 days, and it takes approximately 7 months to clear in most patients [34,35].

In KEYNOTE-059, a phase II study that examined the efficacy of pembrolizumab monotherapy in recurrent or metastatic gastric cancer patients receiving two or more lines of previous chemotherapy, patients who had pembrolizumab as third-line therapy demonstrated superior outcomes compared with those who had it administered as later-line therapy (16.4% versus 6.4%) [25]. In the present study, patients receiving pembrolizumab as third-line therapy achieved a lower response rate compared with those receiving it as later-line therapy (7.0% versus 12.2%; odds ratio 2.297, 95% CI 0.851–6.198, *p* = 0.101).

A positive correlation between HER2 IHC positivity and pathologic types of gastric cancer (intestinal versus diffuse) has been shown in some studies. However, a negative correlation was reported between HER2 IHC positivity and pathologic gastric cancers in other studies [30]. In the present study, 11 of 44 patients (25%) with intestinal or mixed-type gastric cancers were HER2-positive, while just 1 of 69 patients (1.4%) in diffuse or indeterminate-type gastric cancers were HER2-positive. Regarding the relationship between HER2 positivity and PD-L1 positivity, there are conflicting data regarding both positive and negative correlations [35,36]. In this study, there was no correlation between HER2 positivity and PD-L1 positivity. 

This study has several limitations. There were a relatively small number of HER2-positive patients (*n* = 12, 10.6%) among all patients. Although we tried to use a matching algorithm to match HER2-negative and HER2-positive patients at a 2:1 ratio based on age, there was still imbalance after matching. Therefore, our results should be interpreted conservatively. We could not collect the exact data about adverse events (AEs) due to the retrospective nature of the study; if these data were collected together, it would be possible to check the effect of AE on treatment outcomes. 

## 5. Conclusions

In conclusion, pembrolizumab may be more beneficial in HER2-positive gastric cancer patients than in their HER2-negative counterparts. This suggests that HER2 positivity can be a useful predictive biomarker for salvage ICIs in gastric cancer patients.

## Figures and Tables

**Table 1:** Demographic and clinical characteristics of study population.

Study Characteristic	*n* = 113	HER2-Positive (*n* = 12)	HER2-Negative (*n* = 101)	*p*-Value
Age–median (range), years	61 (32–84)	69.5 (50–82)	60 (32–84)	0.005 *
Sex–no. (%)				0.999
Male	73 (64.6)	65	8	
Female	40 (35.4)	36	4	
Performance status–no. (%)				0.999
ECOG 0–1	83 (73.4)	9	74	
ECOG 2–3	30 (26.5)	3	27	
Histologic classification–no. (%)				0.001
Intestinal or mixed	44 (38.9)	11	33	
Diffuse or indeterminate	69 (61.1)	1	68	
PD-L1 status–no. (%)				0.286
CPS ≥ 1	33 (29.2)	2	31	
CPS < 1	12 (10.6)	2	10	
Unavailable	68 (60.2)	8	60	
EBV status–no. (%)				0.999
Positive	3 (2.6)	0	3	
Negative	83 (73.5)	8	75	
Unknown	27 (23.9)	4	33	
MSI status–no. (%)				0.999
Unstable (MSI high)	2 (1.8)	0	2	
Stable	99 (87.6)	9	90	
Unknown	12 (10.6)	3	9	
Prior palliative chemotherapy				0.140
Median (range)	3 (2–5)	3 (2–5)	3 (2–5)	
2–no (%)		3 (25)	49 (48.5)	
≥3–no (%)		9 (75)	52 (51.5)	
Prior anti-HER2 therapy–no. (%)		12 (100%)		

Cooperative Oncology Group; PD-L1, programmed cell death-ligand 1; CPS, combined positive score; EBV, Epstein–Barr virus; MSI, microsatellite instability; *, statistically significant.

**Table 2:** Overall response rate and disease control rate according to HER2 status.

	Overall (*n* = 92)	HER2-Positive (*n* = 10)	HER2-Negative (*n* = 82)	Odds Ratio (95% Confidence Interval)	*p*-Value
Complete response	0	0	0		
Partial response	9	5	4		
Stable disease	29	2	27		
Progressive disease	54	3	51		
					<0.001 *
Overall response rate (%)	9.8	50	4.9	18.1 (2.9–127.0)	<0.001 *
Disease control rate (%)	41.3	70	37.8	3.8 (0.8–24.3)	0.086

*, statistically significant.

## Data Availability

The datasets generated during and/or analyzed during the current study are available from the corresponding author on reasonable request.
